# Association of Chronic Conditions With Bladder Health in Women: Cross-Sectional Results From the RISE FOR HEALTH Study

**DOI:** 10.5888/pcd22.250197

**Published:** 2025-09-25

**Authors:** Camille P. Vaughan, Gerald McGwin, Jean F. Wyman, Siobhan Sutcliffe, Diane K. Newman, Ariana L. Smith, Colleen M. Fitzgerald, Todd Rockwood, James Griffith, Sheila Gahagan, Alayne D. Markland, for the Prevention of Lower Urinary Tract Symptoms (PLUS) Research Consortium

**Affiliations:** 1 Division of Geriatrics and Gerontology, Department of Medicine, Emory University, Atlanta, Georgia; 2 Birmingham/Atlanta VA Geriatric Research Education and Clinical Center, Atlanta, Georgia; 3 Department of Epidemiology, School of Public Health, University of Alabama at Birmingham; 4 School of Nursing, University of Minnesota, Minneapolis; 5 Washington University School of Medicine, Department of Surgery, Division of Public Health Sciences, St. Louis, Missouri; 6 Department of Surgery, Perelman School of Medicine, University of Pennsylvania, Philadelphia; 7 Department of Surgery, Division of Urology, Perelman School of Medicine, University of Pennsylvania, Philadelphia; 8 Department of Obstetrics and Gynecology, Loyola University Chicago, Illinois; 9 Division of Health Policy and Management, School of Public Health, University of Minnesota, Minneapolis; 10 Department of Obstetrics and Gynecology, University of Chicago, Illinois; 11 Division of Academic General Pediatrics, Child Development, and Community Health, Department of Pediatrics, University of California, San Diego; 12 Division of Geriatrics, Department of Internal Medicine, University of Utah, Salt Lake City; 13 Clinical Center at the Birmingham Veterans Affairs Health Care System, Alabama

## Abstract

**Introduction:**

Women with multiple chronic conditions are more likely than women without them to report lower urinary tract symptoms (LUTS); understanding the association of common and coexisting chronic conditions with bladder health across adulthood may inform prevention efforts.

**Methods:**

Data were collected from May 2022 through December 2023 from a regionally representative cohort of community-dwelling adult women in the US. Chronic conditions were assessed by self-report and bladder health, and LUTS were measured using validated questionnaires. Analyses were limited to women aged 40 years or older and included multivariable linear and logistic regression models, adjusted for age, body mass index, physical function, and educational attainment.

**Results:**

Of 3,423 eligible participants, 2,016 were aged 40 years or older and responded to questions on multiple chronic conditions. Of these, 449 had no chronic conditions, 405 reported 1 chronic condition, 652 had 2 or 3 chronic conditions, and 510 had 4 or more chronic conditions. Hypertension (41.4%) and osteoarthritis (13.6%) were the most frequently reported coexisting conditions (9.7% had both). Across the 10-item Bladder Health Scales and 6-item Bladder Function Indices, women with 0 or 1 chronic condition reported better bladder health than women with multiple chronic conditions. In this cohort, frequent LUTS did not vary by the number of chronic conditions.

**Conclusion:**

The opportunity to promote bladder health among adult women with chronic conditions may precede the development of frequent LUTS. Additional research is needed to determine whether prevention strategies may differ according to common chronic conditions.

**Summary**

**What is known on this topic?**

Women with multiple chronic conditions are more likely than women without chronic conditions to report lower urinary tract symptoms (LUTS).

**What is added by this report?**

We demonstrated impaired bladder health with increased number of chronic conditions without finding an increased odds of LUTS across women with multiple chronic conditions. This finding reinforces that bladder health is a distinct measure.

**What are the implications for public health practice?**

Our study presents a unique opportunity to understand potential targets for prevention of LUTS, along with better recognition of bladder health and LUTS in clinical settings and in the community, among women with multiple chronic conditions.

## Introduction

Women with chronic physical and mental health conditions are more likely than women without these conditions to report lower urinary tract symptoms (LUTS), which can substantially affect daily life (1–4). LUTS encompass a range of symptoms related to urine storage and voiding, including frequency, urgency, nocturia, pain, and incomplete bladder emptying. Chronic conditions, either separately or in coexisting clusters, can influence the onset, severity, and progression of LUTS, which then affects overall bladder health and function (1). These conditions include osteoarthritis, diabetes, asthma, chronic obstructive pulmonary disease, cardiovascular diseases such as heart failure and hypertension, obesity, anxiety, depression, and neurological disorders such as stroke and Parkinson disease (1,4–10).

Although strong evidence exists linking individual and multiple chronic conditions to LUTS, their associations with bladder health, a related but broader concept than symptom severity and burden, is not well understood. As defined by the Prevention of Lower Urinary Tract Symptoms (PLUS) Research Consortium, bladder health encompasses total physical, mental, and social well-being related to bladder function and health, not merely the absence of urinary symptoms (11). Cross-sectional studies from the PLUS Research Consortium reveal a wide range of bladder well-being and high use of adaptive/coping behaviors among women with and without urinary symptoms (12). Because the presence of LUTS is associated with worsened quality of life among people living with chronic conditions (13), understanding whether bladder health as a potential target to enhance well-being among those living with common chronic conditions may expand the impact of prevention and health promotion efforts. To our knowledge, no studies have examined the relationship of chronic health conditions with bladder health. The PLUS Consortium’s RISE FOR HEALTH study was designed to investigate factors that promote bladder health and prevent LUTS in community-dwelling women (14).

We used RISE FOR HEALTH data to examine the associations between single and multiple chronic conditions with bladder health and LUTS among middle-aged and older women (14). We described the most common coexisting conditions among women with more than 1 chronic condition. This information has potential for identifying populations at risk of LUTS among those with chronic conditions, improving chronic disease management approaches, and developing LUTS prevention and bladder health promotion strategies.

## Methods

### Study population and design

The RISE FOR HEALTH study, conducted by the PLUS Research Consortium, is an ongoing regionally representative cohort study of US women designed to identify factors positively and negatively associated with bladder health and LUTS across the lifespan. Study design and procedures are described elsewhere (14). Women residing in 1 of 50 counties surrounding 9 university research centers were selected from a marketing database by using simple and stratified probability sampling by age and race and ethnicity to achieve a regionally representative sample. Participants were invited to complete two 30-minute baseline surveys through a web portal; those who did not complete online surveys were mailed paper versions on 2 separate occasions. Eligibility included 1) birth as female or identification as woman, 2) being aged 18 years or older, and 3) the ability to complete the surveys independently in English or Spanish. Individuals consented by completing the initial baseline survey from May 2022 through December 2023. The University of Minnesota Institutional Review Board (IRB) served as the single IRB of record.

The present analysis includes women who participated in the baseline survey and provided complete information on their age and self-reported chronic conditions. To focus on women at highest risk for chronic conditions, only women aged 40 years or older were included. Individuals who did not identify as cisgender women were excluded due to potential anatomic and physiologic alterations in the urinary tract. We did not exclude women who reported having pelvic organ prolapse, congenital anomalies, or previous urogenital surgery. Women who identified as being “currently pregnant” were excluded.

### Chronic condition assessment

Chronic conditions were assessed by using self-reported items asking if a health care provider had ever told the participant that they had any of the listed conditions. The list included 10 items on general medical and psychiatric conditions; 8 items on neurologic conditions; 9 items on bladder, gynecologic, or gastrointestinal conditions; 7 items on cancer conditions; 9 items on prior surgical procedures; and 3 items on bladder treatments. The focus for this analysis included common chronic conditions with a leading role as a cause of death or illness, including diabetes, arthritis, cardiovascular disease, pulmonary disease, and neurologic disease as listed in the Appendix (15). Items were used in their original form (from the National Health and Nutrition Examination Survey or the Boston Area Community Health survey) (16,17) when possible, adapted for use in our study when needed, or created when appropriate existing items did not exist.

### Bladder health assessment

Bladder health was assessed by using the validated PLUS Bladder Health Scales (BHS) and Bladder Function Indices (BFI) (18). Ten scales in the BHS cover the following: 1) global bladder health, 2) holding, 3) urination, 4) social/occupational, 5) physical activity, 6) intimacy, 7) travel, 8) emotion, 9) perception, and 10) freedom. An adaptive behavior adjustment (ABA) is used with the BHS to account for self-reported coping behaviors, such as toilet mapping or use of pads, that affect individual variations in symptom severity and quality of life. The ABA includes use of absorbent products and toilet mapping (19). BFI consists of 6 items that assess the following functions: comfort, frequency, sensation, emptying, continence, and urinary dysbiosis (eg, urinary tract infection). These items were developed and evaluated as indices to assess periodicity, resilience, interference, and relative change in functions.

Scoring for the BHS, BFI, and ABA requires that more than 50% of the items within a scale be completed. The ABA value is the sum of behavior items and confidence indicators associated with each behavior. Adjusted BHS scores were used for analyses and range from 0 to 100, with zero representing the unhealthiest and 100 representing optimal health (18). The 6 items in the BFI were scored as the sum of index items within a domain, from 0 to 100, with higher values indicating better bladder function for that BFI domain. A total BFI score was created by taking the mean of the 6 individual BFI scores. The domains of the BHS and BFI are reported as means (SD), and the possible range is 0 to 100.

### LUTS assessment

LUTS were assessed by using the 10-item Symptoms of Lower Urinary Tract Dysfunction Research Network Symptom Index-10 (LURN SI-10), which is a brief validated instrument that measures the frequency of 10 clinically significant LUTS, including storage, voiding/emptying, and pain with bladder filling within the preceding 7 days; the total score ranges from 0 to 38 (20). Most items are assessed on scales that range from 0 (“never”) to 4 (“every time”), except for daytime (≤3, 4–7, 8–10, ≥11 times per day) and nighttime (0, 1, 2 or 3, >3 times per night) urination frequency. For this analysis, in addition to reporting the total score, we dichotomized symptoms based on women reporting individual LUTS at least 50% or more of the time in the preceding 7 days, daytime frequency 11 times or more per day, or nighttime frequency 2 times or more times per night.

### Covariates

Covariates were selected based on their potential influence on the association between chronic conditions and bladder function. Covariates included age categories based on deciles, body mass index (BMI), physical function as reported by the PROMIS Physical Function standardized score, educational attainment, and cigarette smoking history. For most PROMIS instruments, a score of 50 is the average for the US general population with a SD of 10 because calibration testing was performed on a large sample of the general population (21).

### Statistical analysis

We used descriptive statistics to compare sociodemographic and clinical characteristics across groups defined by self-reported chronic conditions. Separate models regressed BHS and BFI scores by the presence and number of chronic conditions. To describe the distribution of clinically significant LUTS, we reported the proportion of individuals reporting symptoms “about half the time or more.” Daytime and nighttime voiding frequencies were reported based on ordinal categories to differentiate normal frequencies from frequencies that are often associated with increased bother. Linear regression was applied for the BHS and BFI with adjustment for covariates that were either clinically relevant or confounders of the association with chronic conditions. For these analyses, women with 4 or more conditions served as the reference group. We calculated adjusted means for each BHS domain across chronic condition categories from the final linear regression model. We used logistic regression for individual LURN SI-10 symptoms. Multivariable models regressed individual LUTS and chronic condition groups among the total sample. For these analyses, women with no chronic conditions served as the reference group to align with other published literature (1). Smoking status was assessed for inclusion in the full model through a backward selection procedure because it could precede chronic conditions associated with urinary symptoms and bladder health; however, inclusion of smoking status did not change the association between chronic conditions using a 10% change criterion for BHS and BFI scores or LUTS and was not included in the final model. In an exploratory analysis, we applied a stepwise approach to the inclusion of BMI category and physical function score to models evaluating chronic conditions and the BHS global bladder health scale and the total average BFI. We used SAS version 9.4 (SAS Institute Inc) to conduct statistical analyses; *P *< .05 was considered significant by 2-sided *t* test (means) or χ^2^ (proportions) analysis.

## Results

Among 3,423 eligible participants, 2,016 were aged 40 years or older and responded to questions on multiple chronic conditions (Table 1). Of these, 449 (22.3%) had no chronic conditions, 405 (20.0%) had 1 chronic condition, 652 (32.3%) had 2 or 3 chronic conditions, and 510 (25.3%) had 4 or more chronic conditions. Hypertension (41.4%) and osteoarthritis (13.6%) were the most prevalent conditions among participants with 1 chronic condition (Figure) and the most frequently reported coexisting conditions (9.7% had both). Women with multiple chronic conditions were older than women with no chronic conditions or 1 chronic condition and less likely to self-report Asian race. We found no difference by self-reported Hispanic ethnicity. Women with multiple chronic conditions were more likely to be obese and have less than a bachelor’s degree. Parity was significantly associated with the number of chronic conditions, but the direction of the association was inconsistent. Women with 4 or more chronic conditions were more likely to report fair/poor health status and have lower mean (SD) standardized physical function scores (43.2 [9.0]) than women with 0 (56.1 [6.5]) or 1 (53.0 [7.5]) chronic condition (*P* <.001).

**Table 1:** Characteristics of Participants Aged ≥40 Years (N = 2,016), RISE FOR HEALTH Study, United States, 2022–2023^a^

Characteristic	No. (%)				*P* value^c^
	0 Chronic conditions (n = 449)	1 Chronic condition (n = 405)	2 or 3 Chronic conditions (n = 652)^a^	≥4 Chronic conditions (n = 510)^b^	
**Age, mean (SD), y**	54.9 (9.6)	60.2 (10.6)	63.3 (12.1)	66.6 (11.1)	<.001
**Age group, y**					
40–49	150 (32.4)	75 (18.3)	94 (14.2)	34 (6.6)	<.001
50–59	168 (36.3)	111 (27.1)	154 (23.3)	99 (19.2)	
60–69	106 (22.9)	141 (34.5)	199 (30.1)	166 (32.2)	
≥70	39 (8.4)	82 (20.0)	214 (32.4)	216 (41.9)	
**Race**					
Asian	28 (6.2)	33 (8.2)	32 (5.0)	13 (2.6)	<.001
Black or African American	39 (8.7)	56 (14.0)	73 (11.4)	74 (14.7)	
White	313 (69.7)	266 (66.5)	462 (72.3)	355 (70.6)	
Multiple races	3 (0.7)	7 (1.7)	11 (1.7)	10 (2.0)	
Other race^d^	8 (1.8)	3 (0.7)	1 (0.2)	6 (1.2)	
**Ethnicity**					
Hispanic	58 (12.9)	35 (8.7)	60 (9.4)	45 (8.9)	.18
Not Hispanic	398 (87.3)	358 (91.1)	581 (90.6)	454 (91.0)	
**Body mass index**					
Underweight (<18.5 kg/m^2^)	11 (2.5)	9 (2.3)	8 (1.3)	4 (0.8)	<.001
Healthy weight (18.5 to <25 kg/m^2^)	221 (49.7)	152 (39.6)	193 (31.5)	103 (21.5)	
Overweight (25 to <30 kg/m^2^)	91 (20.4)	118 (30.7)	195 (31.8)	115 (24.0)	
Obese (≥30 kg/m^2^)	122 (12.4)	105 (27.3)	217 (35.4)	257 (53.7)	
**Educational attainment**					
High school graduate or less	45 (9.8)	46 (11.3)	88 (13.4)	86 (17.0)	<.001
Some college or vocational training	48 (10.4)	63 (15.4)	120 (18.3)	111 (21.9)	
Vocational or associates degree	69 (15.0)	54 (13.2)	90 (13.7)	85 (16.8)	
Bachelor’s degree	145 (31.4)	116 (28.4)	191 (29.2)	114 (22.5)	
Graduate degree	154 (33.4)	129 (31.6)	166 (25.3)	110 (21.7)	
**Cigarette smoking status**					
Current	18 (3.9)	14 (3.4)	46 (7.1)	29 (5.7)	<.001
Former	106 (23.2)	83 (20.4)	179 (27.4)	185 (36.2)	
Never	332 (72.8)	309 (76.1)	427 (65.4)	297 (58.1)	
**Parity^e^**					
Nulliparous	128 (28.0)	145 (36.6)	242 (37.9)	212 (42.9)	.04
1	59 (12.9)	39 (9.8)	86 (13.5)	61 (12.3)	
2	161 (35.2)	111 (28.0)	172 (26.9)	130 (26.3)	
3	71 (15.5)	74 (18.7)	92 (14.4)	54 (10.9)	
≥4	38 (8.3)	27 (6.8)	47 (7.4)	37 (7.5)	
**Mode of delivery**					
Never pregnant	128 (28.6)	145 (36.6)	242 (37.9)	212 (42.9)	.15
Vaginal delivery only	231 (51.7)	171 (43.5)	273 (43.3)	191 (39.7)	
Cesarean delivery only	54 (12.1)	45 (11.4)	78 (12.4)	51 (10.6)	
**General health status**					
Excellent	127 (27.7)	70 (17.2)	78 (11.9)	20 (3.9)	<.001
Very good	236 (51.3)	228 (56.0)	327 (49.7)	159 (31.1)	
Good	85 (18.5)	96 (23.6)	213 (32.4)	228 (44.6)	
Fair/poor	12 (2.6)	13 (3.2)	36 (5.5)	104 (20.3)	
PROMIS Physical Function, mean standardized score (SD)^f^	56.1 (6.5)	53.0 (7.5)	49.2 (8.3)	43.2 (9.0)	<.001
**Comorbidity^g^**					
Hypertension	NA	168 (41.4)	366 (55.4)	123 (23.9)	<.001
Diabetes	NA	22 (5.4)	111 (16.8)	161 (31.7)	
Osteoarthritis	NA	55 (13.6)	228 (34.8)	362 (71.7)	
Chronic obstructive pulmonary disease	NA	35 (8.6)	100 (15.3)	179 (35.1)	
Depression	NA	34 (8.4)	179 (27.4)	225 (44.4)	
Anxiety	NA	40 (9.9)	183 (28.2)	187 (37.0)	
Sleep apnea	NA	10 (2.5)	60 (9.4)	180 (36.7)	
Cerebrovascular accident	NA	2 (0.5)	13 (2.0)	24 (4.7)	

Abbreviation: NA, not applicable.

a Of participants with 2 conditions, 9.7% had hypertension and osteoarthritis; 8.1% had anxiety and depression; and 5.9% had hypertension and diabetes. Of participants with 3 conditions, 3.4% had hypertension, osteoarthritis, and inflammatory arthritis; 2.4% had hypertension, osteoarthritis, and sleep apnea.

b Of participants with 4 conditions, 1.9% had hypertension, diabetes, osteoarthritis, and sleep apnea.

c Determined by *t* test (means) or χ^2^ test (proportions); *P* value < .05 considered significant.

d Includes American Indian or Alaska Native (n = 4), Ashkenazic (n = 1), Middle Eastern or North African (n = 8), Native Hawaiian or Other Pacific Islander (n = 3), and unspecified other race (n = 2).

e Excludes pregnant now.

f Raw scores (range, 10–50) are converted to a *t* score that rescales the raw score into a standardized score with a mean of 50 and an SD of 10, with higher scores indicating better physical function.

g In addition to the conditions listed, the survey asked about congestive heart failure or coronary artery disease, pulmonary disease, inflammatory arthritis, kidney failure, and neurologic diseases. Neurologic diseases included the following conditions: mild cognitive impairment or memory loss (n = 46); multiple sclerosis (n = 19); Parkinson disease (n = 6); spina bifida (n = 3); spinal cord injury or brain injury, including traumatic brain injury (n = 16); spinal stenosis, spinal disc disease, spinal nerve damage, or sciatica (n = 240); stroke (n = 39); and transient ischemic attack (n = 44). Two participants reported being on dialysis, and 258 participants reported having inflammatory arthritis. Additional details on conditions are provided in the Appendix.

**Figure:**
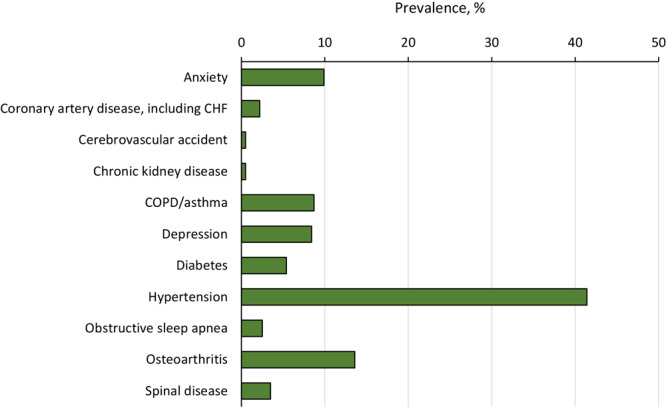
Prevalence of chronic conditions among RISE FOR HEALTH study participants aged 40 years or older with 1 chronic condition. Abbreviations: CHF, congestive heart failure; COPD, chronic obstructive pulmonary disease.

**Table:** 

Condition	Prevalence, %
Anxiety	9.9
Coronary artery disease, including CHF	2.2
Cerebrovascular accident	0.5
Chronic kidney disease	0.5
COPD/asthma	8.7
Depression	8.4
Diabetes	5.4
Hypertension	41.4
Obstructive sleep apnea	2.5
Osteoarthritis	13.6
Spinal disease	3.5

Across multiple scales of the BHS (range, 0–100) and BFI (range, 0–100), women with 0 or 1 chronic condition reported better bladder health than women with multiple chronic conditions (2 or 3 or ≥4 conditions) after adjustment (Table 2). The physical activity-related bladder health scale was not associated with the presence of chronic conditions. On the BHS global bladder health scale, women with 0 or 1 chronic condition had higher scores (scores, 56.4 and 55.9, respectively) than women with 4 or more chronic conditions (score, 51.4). The total average score for the BFI was lower among women with 4 or more chronic conditions (score, 70.3) than among women in any other category; it was 4.9 points lower among women with no chronic conditions (score, 75.2). The number of chronic conditions or lack of chronic conditions was not associated with the BFI’s continence item.

**Table 2:** Adjusted^a^ Mean Scores of Adapted Bladder Health Scales and Bladder Function Indices Among Participants Aged ≥40 Years (N = 2,016), by Number of Chronic Conditions, RISE FOR HEALTH Study, United States, 2022–2023

Scale or index	Mean (95% CI)			
	0 Chronic conditions (n = 449)	1 Chronic condition (n = 405)	2 or 3 Chronic conditions (n = 652)	≥4 Chronic conditions (n = 510)
**Bladder Health Scales^b^**				
Global bladder health	56.4 (52.6–60.3)^c^	55.9 (52.0–59.7)^c^	53.1 (49.6–56.6)	51.4 (47.4–55.4)
Holding	60.1 (56.1–64.0)^c^	59.2 (55.2–63.2)^c^	57.0 (53.3–60.6)	54.6 (50.4–58.8)
Urination	65.7 (62.0–69.4)^c^	65.1 (61.4–68.9)^c^	61.6 (58.2–65.0)	58.8 (54.9–62.7)
Social/occupational	69.8 (65.8–73.7)^c^	69.6 (65.7–73.6)^c^	67.0 (63.4–70.7)	64.6 (60.4–68.7)
Physical activity	64.7 (60.5–68.9)	63.6 (59.3–67.9)	62.5 (58.6–66.4)	60.3 (55.9–64.8)
Intimacy	70.1 (66.0–74.3)^c^	70.1 (65.9–74.3)^c^	66.5 (62.7–70.3)	64.4 (59.9–68.8)
Travel	65.9 (61.8–70.0)^c^	64.8 (60.6–68.9)^c^	62.4 (58.7–66.2)	59.9 (55.6–64.2)
Emotion	65.3 (61.2–69.5)^c^	65.4 (61.2–69.6)^c^	61.9 (58.1–65.7)	59.7 (55.3–64.1)
Perception	65.3 (61.2–69.4)^c^	64.0 (59.8–68.1)^c^	61.6 (57.8–65.4)	58.2 (53.8–62.5)
Freedom	62.7 (58.7–66.7)^c^	61.5 (57.5–65.6)	59.4 (55.7–63.1)	57.5 (53.3–61.8)
**Bladder Function Indices^d^**				
Total average score	75.2 (72.8–77.6)^c^	74.1 (71.7–76.5)^c^	72.7 (70.5–74.9)^c^	70.3 (67.8–72.8)
Comfort	83.0 (79.5–86.4)^c^	80.4 (76.9–83.9)^c^	81.8 (78.6–85.0)^c^	75.5 (71.8–79.1)
Frequency	69.0 (65.2–72.8)^c^	67.8 (64.0–71.6)^c^	64.8 (61.3–68.3)	63.3 (59.4–67.3)
Sensation	69.4 (65.9–73.0)^c^	67.2 (63.6–70.8)	65.3 (62.0–68.6)	63.5 (59.8–67.3)
Emptying	71.5 (67.6–75.4)	72.8 (68.8–76.8)^c^	70.4 (66.8–74.1)	68.2 (64.1–72.4)
Continence	64.7 (61.6–67.9)	61.9 (58.7–65.1)	61.9 (59.0–64.8)	61.9 (58.6–65.2)
Biosis/UTI history	93.0 (90.2–95.8)^c^	95.0 (92.2–97.9)^c^	90.9 (88.3–93.5)	89.1 (86.1–92.0)

Abbreviation: UTI, urinary tract infection.

a Adjusted for age group (40–49, 50–59, 60–69, ≥70 y); body mass index (4 categories), PROMIS Physical Function (continuous), and educational attainment (5 categories).

b Scores range from 0 to 100, with zero representing the unhealthiest and 100 representing optimal health (18).

c Indicates a significant difference in the mean (95% CI) compared with the reference group (≥4 chronic conditions).

d Scores range from 0 to 100, with higher values indicating better bladder function for that domain (18).

Overall, less than 10% of the cohort reported urgency and less than 10% reported stress urinary incontinence at least 50% of the time in the past week. After adjustment for confounding factors, the total LURN SI-10 score and the odds of clinically significant LUTS did not vary by the number of chronic conditions (Table 3). In an exploratory analysis of stepwise inclusion of BMI and physical function score after adjusting for age and education, the difference in the BHS global bladder health score between the group with 0 chronic conditions (score, 60.5) and the group with 4 or more chronic conditions (score, 42.9) was 17.6 points without including BMI or physical function. The difference narrowed to 14.0 points after including BMI (score, 60.7 with 0 chronic conditions; score, 46.7 with ≥4 chronic conditions) and further narrowed to the 5.0-point difference observed in the full model, which included the physical function score (score, 56.4 with 0 chronic conditions; score, 51.4 with ≥4 chronic conditions). For the total average BFI score, the difference between the group with 0 chronic conditions (score, 79.8) and the group with 4 or more chronic conditions (score, 67.1) was 12.7 points without including BMI or physical function. The difference narrowed to 11.6 points after including BMI (score, 78.3 with 0 chronic conditions; score, 66.7 with ≥4 chronic conditions) and further narrowed to the 4.9-point difference observed in the full model, which included the physical function score (score, 75.2 with 0 chronic conditions; score, 70.3 with ≥4 chronic conditions).

**Table 3:** LURN Symptoms Reported Most or Every Time Among Participants Aged ≥40 Years (N = 2,016), RISE FOR HEALTH Study, United States, 2022–2023^a^

Symptom	0 Chronic conditions, no. (%) (n = 449)	1 Chronic condition (n = 405)		2 or 3 Chronic conditions (n = 652)		≥4 Chronic conditions (n = 510)	
		No. (%)	OR^b^ (95%CI)	No. (%)	OR^b^ (95%CI)	No. (%)	OR^b^ (95%CI)
Urgency	21 (4.6)	32 (7.9)	1.29 (0.64–2.59)	64 (9.8)	1.10 (0.58– 2.10)	73 (14.3)	1.07 (0.53–2.17)
Urge urinary incontinence	16 (3.5)	21 (5.2)	0.99 (0.45–2.19)	52 (7.9)	1.04 (0.51–2.12)	62 (12.2)	1.16 (0.54–2.51)
Stress urinary incontinence (laugh, sneeze, cough)	23 (5.0)	29 (7.1)	1.14 (0.60–2.19)	64 (9.7)	1.35 (0.75–2.42)	46 (9.0)	1.03 (0.52–2.05)
Stress urinary incontinence (physical activity)	13 (2.8)	15 (3.7)	1.17 (0.51–2.72)	35 (5.4)	1.00 (0.46–2.21)	35 (6.9)	1.08 (0.45–2.59)
Pain while filling	1 (0.2)	5 (1.2)	1.94 (0.16–22.9)	6 (0.9)	2.09 (0.22–19.84)	7 (1.4)	1.32 (0.12–15.15)
Delay before urine starts	3 (0.7)	4 (1.0)	1.79 (0.29–11.11)	12 (1.8)	2.26 (0.44–11.52)	15 (2.9)	3.72 (0.69–20.12)
Slow or weak urine	9 (2.0)	8 (2.0)	0.90 (0.29–2.79)	20 (3.0)	0.67 (0.24–1.89)	28 (5.5)	1.11 (0.39–3.25)
Dribbling after zipping or pulling up underwear	11 (2.4)	14 (3.5)	0.63 (0.21–1.86)	22 (3.4)	0.74 (0.30–1.83)	27 (5.3)	0.97 (0.36–2.60)
Daytime voiding 11 times or more	24 (5.5)	104 (26.9)	1.00 (0.44–2.26)	153 (25.0)	0.77 (0.35–1.69)	119 (24.9)	0.93 (0.39–2.23)
Nighttime voiding 2 times or more	17 (3.7)	117 (28.7)	0.34 (0.14–0.87)	252 (38.4)	0.56 (0.28–1.13)	222 (43.5)	0.65 (0.30–1.41)

Abbreviations: LURN, Symptoms of Lower Urinary Tract Dysfunction Research Network; OR, odds ratio.

a Mean (95% CI) LURN Symptom Index-10 scores were the following for each group: 5.7 (5.0–6.4) for 0 chronic conditions; 5.9 (5.1–6.6) for 1 chronic condition; 5.8 (5.2–6.5) for 2 or 3 chronic conditions; 5.8 (5.1–6.6) for ≥4 chronic conditions. The total score for the scale can range from 0 to 38, with higher scores indicating more frequent urinary symptoms.

b Adjusted for age group (40–49, 50–59, 60–69, ≥70 y); body mass index (4 categories), PROMIS Physical Function score (continuous), and education (5 categories). The reference group for all odds ratios was the group with 0 chronic conditions.

## Discussion

Women enrolled in the RISE FOR HEALTH study were selected to inform understanding about bladder health and generally had fewer LUTS compared with women in other studies, although 69% of participants reported using adaptive/coping behaviors, including using pads (40%), toilet mapping (58%), and staying close to a toilet (3%) (12). Women participating in RISE FOR HEALTH were also not likely to discuss LUTS with a clinical provider: only 38% had discussed LUTS, similar to the percentage in other studies (12,22). We demonstrated impaired bladder health with an increased number of chronic conditions without finding an increased odds of LUTS (both individual LUTS and as a global score) across women with multiple chronic conditions. This finding reinforces that bladder health is a distinct measure. Thus, our study presents a unique opportunity to understand potential targets for prevention of LUTS, along with better recognition of bladder health and LUTS in clinical settings and in the community, among women with multiple chronic conditions.

Compared with previous studies of chronic conditions and LUTS in women, our study found similar types and prevalence of chronic conditions among women aged 40 or older in the RISE FOR HEALTH cohort. We observed that the most common types of chronic conditions were hypertension and osteoarthritis, similar to observations in other cross-sectional population-based studies (1,4,23,24). We also found prevalence estimates of depression, anxiety, and diabetes that were similar to estimates in other studies among women with LUTS. In contrast, we did not observe pulmonary diseases, such as asthma, as a distinct condition associated with change in bladder health in RISE FOR HEALTH. Unique to RISE FOR HEALTH, we described patterns of chronic conditions associated with bladder health in women, rather than focusing on LUTS, as our primary outcome. Thus, we believe our study provides further evidence that the concept of bladder health is distinctive and offers data that can be compared with the data of other studies that evaluate the association between LUTS and chronic conditions among women. In general, asking about bladder health among women, especially women with chronic conditions who are seeking medical care for these conditions, could be important for prevention and treatment of LUTS.

Osteoarthritis cause pain and stiffness that can lead to muscle weakness, which in turn may impair mobility, hinder individuals from reaching the toilet, and can lead to urinary incontinence and exacerbate other LUTS (10). The frequency of osteoarthritis and reduction in physical function among RISE FOR HEALTH participants with multiple chronic conditions highlights the intersection of mobility and bladder health. Other studies have also reported associations between physical function and mobility among women with LUTS (25,26). Our exploratory analysis further bolsters the observation that physical function contributes to the association between multiple chronic conditions and bladder health. Additional studies focused on bladder health among people who are engaged in more physical function and activity interventions may be an early target for prevention.

Cardiometabolic disease with coexisting hypertension and diabetes emerged as a common set of coexisting chronic conditions among those with 2, 3, or 4 or more conditions, which has been observed previously among women with LUTS (1). Bladder dysfunction associated with diabetes can increase risk for urinary incontinence, overactive bladder, urinary tract infections, and other urinary symptoms such urgency, frequency, nocturia, and bladder emptying (7,8). Similarly, Parkinson disease is linked with bladder dysfunction, causing LUTS at any stage and worsening as the disease progresses (9). Stroke can cause LUTS due to neurological damage to bladder control and mobility impairments that affect toileting ability. Potential opportunities for understanding underlying mechanisms that contribute to cardiometabolic and neurologic diseases, along with better screening for LUTS in these populations, could contribute to prevention and treatment efforts.

### Strengths and limitations

Strengths of the current study include a sample of adult women across mid-life and late-life ages representative of the regional demography that reflects much of the US population. Additionally, coupling a standardized measure of physical function in the RISE FOR HEALTH assessment with the presence of chronic conditions allowed us to perform adjusted analyses that incorporated a measure of mobility into the modeling strategy. A limitation was the cross-sectional design, which does not permit an assessment of temporality. Data collection for a longitudinal phase of the RISE FOR HEALTH study is in progress, which will permit an evaluation of how the association between chronic conditions and bladder health evolves over time and whether chronic conditions precede the onset of poor bladder health and LUTS. It is possible that self-reported status of chronic conditions could lead to misclassification. The minimum important difference for the BHS and BFI has not been established, and so we are not able to determine whether significant associations between the burden of chronic conditions and bladder health and function are clinically meaningful. In the overall RISE FOR HEALTH cohort, the adjusted mean (SD) BHS global bladder health score was 55 (26); in the subgroup without LUTS, the adjusted mean (SD) BHS global bladder health score was 78 (17). Similarly, the total average score for the BFI was 75 (18) in the overall cohort and 91 (9) in the subgroup without LUTS (12). Thus, the differences observed in the current evaluation of the BHS and BFI across chronic condition groups are not as widely distributed as the differences in the overall cohort.

### Conclusions

Findings from the RISE FOR HEALTH study suggest that bladder health is impaired among adult women with multiple chronic conditions and may precede the development of frequent LUTS. In this cohort, conditions affecting musculoskeletal health, in addition to cardiometabolic disease, were prominent among women with chronic conditions. Additional research is needed to determine whether LUTS prevention strategies and the promotion of bladder health strategies may differ among women with multiple chronic conditions.

## Appendix Assessment of Chronic Conditions in the RISE FOR HEALTH Study, United States, 2022–2023

Response options for all questions were yes and no.

E8. Has a doctor or other health care provider ever told you that you had any of the following conditions?

a. Diabetes (also called sugar diabetes or sugar; not including gestational diabetes or diabetes during pregnancy)
b. High blood pressure or hypertension
c. A heart condition, such as angina, heart attack or myocardial infarction (MI), congestive heart failure (CHF), or “fluid on the lungs”
d. Chronic bronchitis, COPD, emphysema, or asthma
e. Osteoarthritis (wearing down of cartilage in the joints)
f. Inflammatory arthritis (rheumatoid arthritis or psoriatic arthritis)
g. Sleep apnea
h. Kidney failure
h1. If yes; Are you currently being treated by dialysis?
i. Diagnosis or being treated for anxiety, such as panic attacks, social anxiety, obsessive-compulsive behavior, or a phobia
j. Diagnosis or being treated for depression

E9. Has a doctor or other health care provider ever told you that you had any of the following neurological conditions?

Stroke, cerebrovascular accident
TIA, also called transient ischemic attack or ministroke or warning stroke
Multiple sclerosis
Parkinson’s disease
Brain or spinal cord injury (includes traumatic brain injury)
Spina bifida
Spinal stenosis, spinal disc disease, spinal nerve damage, or sciatica
Mild cognitive impairment or memory loss
